# Efficacy of Acetic Acid against *Listeria monocytogenes* Attached to Poultry Skin during Refrigerated Storage

**DOI:** 10.3390/foods3030527

**Published:** 2014-09-11

**Authors:** Elena Gonzalez-Fandos, Barbara Herrera

**Affiliations:** Technology Department, CIVA Research Center, University of La Rioja, Madre de Dios 51, 26006 Logroño, La Rioja, Spain; E-Mail: anabarbaraherrera@redfarma.org

**Keywords:** poultry, decontamination, meat safety, carcass, pathogen reduction, organic acids, *Listeria monocytogenes*

## Abstract

This work evaluates the effect of acetic acid dipping on the growth of *L*. *monocytogenes* on poultry legs stored at 4 °C for eight days. Fresh inoculated chicken legs were dipped into either a 1% or 2% acetic acid solution (v/v) or distilled water (control). Changes in mesophiles, psychrotrophs, Enterobacteriaceae counts and sensorial characteristics (odor, color, texture and overall appearance) were also evaluated. The shelf life of the samples washed with acetic acid was extended by at least two days over the control samples washed with distilled water. *L*. *monocytogenes* counts before decontamination were 5.57 log UFC/g, and after treatment with 2% acetic acid (Day 0), *L*. *monocytogenes* counts were 4.47 log UFC/g. Legs washed with 2% acetic acid showed a significant (*p* < 0.05) inhibitory effect on *L*. *monocytogenes* compared to control legs, with a decrease of about 1.31 log units after eight days of storage. Sensory quality was not adversely affected by acetic acid. This study demonstrates that while acetic acid did reduce populations of *L*. *monocytogenes* on meat, it did not completely inactivate the pathogen. The application of acetic acid may be used as an additional hurdle contributing to extend the shelf life of raw poultry and reducing populations of *L*. *monocytogenes*.

## 1. Introduction

Meat and poultry products are often identified as the source of foodborne pathogens [1]. Raw poultry is a well-recognized source of *L. monocytogenes*, and many surveys have confirmed the presence of this pathogen on fresh poultry [2,3,4]. Some authors have associated cases of listeriosis with the consumption of undercooked chicken [5].

The contamination of raw chicken with bacterial pathogens has important implications for public health. The reduction of poultry contamination with foodborne pathogens during slaughter is particularly important. Since hygienic practices during slaughter cannot completely prevent the contamination of poultry carcasses, decontamination treatments are gaining increasing interest in the slaughter process [6,7,8,9].

Organic acids (acetic, lactic, propionic and sorbic) are increasingly used in food products as preservatives, because of their antibacterial activity, and they occur naturally in foods. Organic acids are generally recognized as safe substances (GRAS) by the FDA and are approved as food additives by European Commission, FAO/WHO and FDA [10].

High concentrations of organic acids are required to be effective as decontaminating agents, but it is important to consider the effect of high concentrations of acids on product quality, since some alterations in the visual appearance of carcasses have been reported [6,10]. Generally, treatments with organic acids at varying concentrations result in population reductions ranging from one to three log units on meat surfaces [7,8,9].

Acetic acid has been investigated as an antimicrobial agent for use in meat, including poultry, to extend its shelf-life and inhibit the growth of pathogens, such as *Salmonella* or *Escherichia coli* [11,12,13,14,15].

The effectiveness of acetic acid for controlling meat-borne pathogens varies between studies and may be attributable to differences in acid concentration, as well as methods for acid delivery, the temperature of acids, contact time, sampling techniques, tissue type or organisms [16].

The ability of acetic acid to inhibit *L. monocytogenes* has been studied in laboratory media [17,18,19,20] and in beef and sheep [21]. However, there are few studies on the effect of acetic acid on *Listeria monocytogenes* growth on poultry [6].

The aim of this work was to evaluate the effectiveness of an acetic acid dip to control the growth of *Listeria monocytogenes* on poultry stored at 4 °C. Microbiological and sensorial quality were also evaluated.

## 2. Experimental Section

### 2.1. Preparation of Bacterial Inoculum

The *Listeria monocytogenes* serotype 1/2a strain CECT 932 was grown in tryptone soya broth (Oxoid, Hampshire, UK) at 30 °C for 18 h to achieve a viable cell population of 9 log CFU/mL. The culture was then transferred to a sterile centrifuge bottle and centrifuged at 10,000× *g* for 10 min at 4 °C. The supernatant was decanted and the pellet resuspended in sterile 0.1% peptone solution (Merck, Darmstadt, Germany) (pH 6.2) by vortexing. The washing step was repeated twice. The suspension of washed cells was diluted in a sterile 0.1% peptone solution to obtain an appropriate cell concentration for inoculation of sterile distilled water.

### 2.2. Inoculation of Poultry and Treatment

Ninety fresh chicken legs were obtained from a poultry processing plant (La Rioja, Spain). The legs were placed on crushed ice and transported to the laboratory.

Fresh chicken legs were inoculated with *L. monocytogenes* by dipping them into a suspension of this pathogen (7 log CFU/mL) for 5 min at room temperature. After the inoculation, the legs were removed and kept for 30 min at room temperature to allow the attachment of inoculated cells to the skin.

The inoculated poultry legs were divided into three groups, each containing 30 legs. Samples of each group were dipped for 5 min into sterile distilled water (control) (group one), 1% (v/v) (group 2) or 2% (group 3) acetic acid (Scharlau, Barcelona, Spain). After these treatments, the legs were removed and drained for 5 min and stored individually in sterile bags left open at 4 °C for 8 days. All experiments were carried out in duplicate.

Samples were taken on Days 0 (after dipping treatment), 1, 3, 6 and 8. On the sampling days, six legs of each group were taken out from storage to perform microbiological, pH and sensorial analysis.

### 2.3. Sensorial Analysis

The samples were evaluated for overall acceptability with regard to odor, color, texture and overall appearance by a panel of 9 members who were regular consumers of poultry meat. A structured hedonic scale [22] with numerical scores ranging from 7 (I like it very much) to 1 (I dislike it very much) was used. A score of 3 was considered the borderline of acceptability [7].

### 2.4. Microbiological Analyses and pH Determination

Ten grams of skin were aseptically weighed and homogenized in a Stomacher (IUL, Barcelona, Spain) for 2 min with 90 mL of sterile peptone water (Oxoid). Further decimal dilutions were made with the same diluent.

Studies were carried out to relate the weight with the surface of the poultry legs. It was found that 1 g of leg skin corresponded to an average of 6.88 cm^2^ of leg skin.

The total number of mesophilic microorganisms was determined on Plate Count Agar (PCA, Merck) following the pour plate method, incubating at 30 °C for 72 h [23]. Psychrotrophs were determined on Plate Count Agar (Merck) with an incubation temperature of 7 °C for 10 days, using the pour plate method [23]. Enumeration of Enterobacteriaceae was carried out on violet red bile glucose (VRBG) (Merck) following the pour plate method with an incubation temperature of 37 °C for 48 h [23]. *Listeria* spp. were determined following the surface plate method on Palcam agar with an incubation temperature of 30 °C for 48 h [24]. Ten suspected colonies grown on Palcam agar were subcultured for purity on tryptone soya agar (TSA) (Merck) and incubated for 24 h at 30 °C. The following identification tests for *L*. *monocytogenes* were performed: Gram stain, catalase reaction, oxidase test, tumbling motility at 20–25 °C, umbrella motility in the SIM medium (Oxoid, Hampshire, UK) and CAMP test [25]. Five suspected isolates were also identified by using API *Listeria* strips (BioMérieux, Marcy l’Etoile, France). The percentage of colonies identified as *L*. *monocytogenes* was 99%.

For pH determination, 5 g of skin were blended with 10 mL of distilled water. The pH of the homogenized sample was measured with a Crison model 2002 pHmeter (Crison Instruments, Barcelona, Spain).

### 2.5. Statistical Analysis

For microbiological data, an analysis of variance was performed using the SYSTAT program for Windows; Statistics version 5.0 (Evanston, IL, USA). Tukey’s test for the comparison of means was performed using the same program. Plate count data were converted to logarithms prior to their statistical treatment. All experiments were carried out in duplicate. The significance level was defined at *p* < 0.05.

The data obtained from sensorial evaluation on the various sampling days were compared for statistical significance using Wilcoxon’s matched pair test. To compare the data obtained on the same day with different concentrations of acetic acid, a Mann-Whitney *U* test was used. The significance level was defined at *p* < 0.05. The tests were carried out using the Statistica 6.0 program (Statsoft, IL, USA).

## 3. Results and Discussion

### 3.1. Microbiological Quality

The effect on mesophiles and psychrotrophs of dipping the legs into different acetic acid concentrations is shown in Table 1 and Table 2, respectively. Significant differences (*p* < 0.05) in mesophile counts were found between the legs treated with 1% or 2% acetic acid and the control legs. The data obtained showed that a 5-min dip in 2% v/v acetic acid reduced mesophiles counts between 1.1 and 2.66 log cycles compared to the control legs throughout storage. After treatment, mesophile counts were about 0.89 or 1.1 log lower than in control samples, depending on the acetic acid concentration. Treatment with 1% or 2% acetic acid extended the lag phase; no growth was observed on Day 1. After six days, mesophile counts on samples treated with 1% or 2% acetic acid were 1.9 and 2.34 log units lower compared to control samples, respectively. Significant differences (*p* < 0.05) were found for these bacterial counts between the samples treated with 1% acetic acid and those treated with 2% acetic acid only on Days 6 and 8, although lower counts were observed in samples treated with 2% acetic acid on the other days.

Significant differences (*p* < 0.05) in psychrotroph counts were found between the legs treated with 1% or 2% acetic acid and the control legs. The dipping with 2% acetic acid reduced psychrotroph counts between 0.49 and 2.43 log cycles compared with the control legs throughout storage.

The results obtained agree with those reported by Fabrizio *et al*. [26], who found that immersion of chicken carcass in 2% acetic acid reduced the total counts between 1.25 and 3.3 log cycles compared to the control legs throughout storage. Dickens and Whittemore [12] also observed that the dipping of poultry carcass into 1% acetic acid reduced mesophiles by 0.6 log cycles. Similar reductions on mesophiles counts were reported by Dickens and Whittemore [11] and Dickens *et al*. [27].

**Table 1:** The effect of acetic acid on mesophile counts on poultry legs (log CFU/g).

Batch	Days of Storage				
	0	1	3	6	8
Control	5.57 ± 0.13 ^a^	7.13 ± 0.04 ^a^	7.69 ± 0.44 ^a^	9.83 ± 0.03 ^a^	10.31 ± 0.01 ^a^
1% Acetic acid	4.68 ± 0.01 ^b^	4.68 ± 0.06 ^b^	6.29 ± 0.03 ^b^	7.93 ± 0.03 ^b^	8.84 ± 0.03 ^b^
2% Acetic acid	4.47 ± 0.01 ^b^	4.47 ± 0.02 ^b^	5.87 ± 0.02 ^b^	7.49 ± 0.01 ^c^	8.31 ± 0.01 ^c^

Notes: Mean ± standard deviation. Means within columns followed by the same letter were not significantly different (*p* > 0.05).

**Table 2:** The effect of acetic acid on the psychrotroph counts on poultry legs (log CFU/g).

Batch	Days of Storage				
	0	1	3	6	8
Control	5.17 ± 0.03 ^a^	5.62 ± 0.01 ^a^	6.88 ± 0.01 ^a^	9.02 ± 0.03 ^a^	9.57 ± 0.01 ^a^
1% Acetic acid	4.87 ± 0.01 ^b^	4.91 ± 0.01 ^b^	5.72 ± 0.03 ^b^	6.77 ± 0.03 ^b^	8.12 ± 0.04 ^b^
2% Acetic acid	4.68 ± 0.02 ^c^	4.69 ± 0.03 ^b^	5.63 ± 0.01 ^b^	6.59 ± 0.01 ^c^	7.89 ± 0.01 ^c^

Notes: Mean ± standard deviation. Means within columns followed by the same letter were not significantly different (*p* > 0.05).

Acetic acid has been also applied to pig, lamb and beef carcasses, being found effective in reducing microbial counts by one log cycle [28,29,30].

In the present study, the treatment with 1% or 2% acetic acid extended the lag phase. Furthermore, Jiménez *et al*. [31] observed that the immersion of chicken breast in a 1% acetic acid solution extended the lag phase of microbial growth.

According to Gill and Landers [32], decontaminating treatments must be regarded as trivial when the numbers of bacteria recovered before and after a treatment do not differ by a minimum of 0.5 log units. In consequence, in the present study, acetic acid treatments could be considered as effective.

In a previous work, it was observed that a treatment with 2% lactic acid reduced mesophile counts between 0.67 and 2.32 log cycles compared with the control legs throughout storage [33]. In the present work, a treatment with 2% acetic acid reduced mesophile counts between 1.1 and 2.66 log cycles. Thus, the antimicrobial effect of acetic acid was higher than lactic acid, if we compare the percentage added. The antimicrobial effect of citric acid was lower than acetic and lactic acids, since a treatment with 2% citric acid reduced mesophile counts between 0.45 and 1.08 log cycles [34].

Other authors have reported that the efficacy of acetic acid was lower than that reached with lactic acid. Thus, Sakhare *et al*. [15] reported that lactic acid was superior to acetic acid as the decontaminating agent to reduce the microbial load on poultry carcasses at different processing steps.

Table 3 shows the effect of acetic acid treatment on the growth of Enterobacteriaceae. Significant differences (*p* < 0.05) in the Enterobacteriaceae counts were observed on legs treated with 1% or 2% acetic acid compared to the control samples. After treatment, Enterobacteriaceae counts were 1.61 log cycles lower in legs treated with 2% acetic acid than in control ones. Significant differences (*p* < 0.05) were also found between the legs treated with 1% acetic acid and those treated with 2% acetic acid on Days 0 and 8.

**Table 3:** The effect of acetic acid on the Enterobacteria counts on poultry legs (log CFU/g).

Batch	Days of Storage				
	0	1	3	6	8
Control	3.04 ± 0.04 ^a^	4.21 ± 0.06 ^a^	5.09 ± 0.01 ^a^	5.70 ± 0.07 ^a^	6.54 ± 0.08 ^a^
1% Acetic acid	2.13 ± 0.01 ^b^	2.64 ± 0.01 ^b^	3.95 ± 0.01 ^b^	4.55 ± 0.01 ^b^	5.51 ± 0.04 ^b^
2% Acetic acid	1.63 ± 0.04 ^c^	2.60 ± 0.01 ^b^	3.84 ± 0.11 ^b^	4.34 ± 0.07 ^b^	5.04 ± 0.03 ^c^

Notes: Mean ± standard deviation. Means within columns followed by the same letter were not significantly different (*p* > 0.05).

In the present study, Enterobacteriaceae counts of treated samples were significantly lower than in control samples. These findings agree with those reported by Dickens and Whittemore [11], who observed that the dipping of poultry carcass into a 0.6% acetic acid solution reduced Enterobacteriaceae counts by 0.71 log cycles. After six days of storage, we observed that Enterobacteriaceae counts on treated samples were lower (about 1.15–1.36 log units) than in control samples. Furthermore, Jiménez *et al*. [31] reported an Enterobacteriaceae reduction of about 1.5 log units in carcasses treated with acetic acid.

### 3.2. pH Evolution

**Figure 1:**
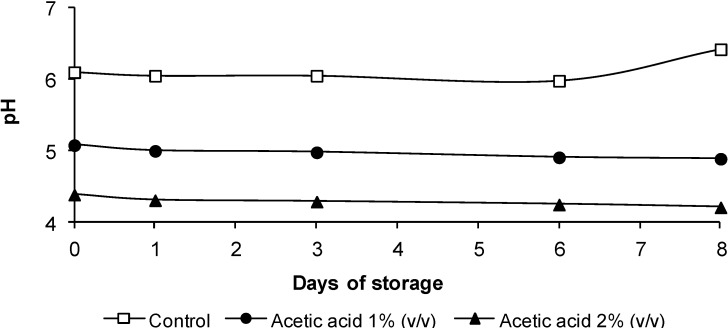
The evolution of pH in chicken legs treated with acetic acid. The data are the mean values of six replicates.

The pH values of the legs treated with acetic acid are shown in Figure 1. Significant differences (*p* < 0.05) were found in pH values between samples treated with 1% or 2% acetic acid and control samples. The pH was lower when the acetic acid concentration was higher. These pH differences did not decrease throughout storage. Initial pH values in legs treated with 2% acetic acid (Day 0) were 4.39 ± 0.04, 1.71 units lower than in control legs.

The pH data indicated that the reductions of bacterial populations may have been due to the effects of acidic pH. Thus, lower counts were observed in legs with lower pH. The antimicrobial effect of organic acids has been attributed to undissociated acid molecules that interfere with cellular metabolism or a decrease in biological activity, as a result of pH changes in the cell’s environment [35,36]. In this study, the application of acetic acid reduced the surface pH immediately after treatment, thereby creating an unfavorable environment for bacterial growth. The mean skin pH value of untreated samples was 6.1. Treatment with 1% or 2% acetic acid solution resulted in a decrease in pH of about one and 1.5 units, respectively. A similar pH decrease in chicken breast after dipping with 1% acetic acid has been reported by Jimenez *et al*. [31].

### 3.3. Listeria monocytogenes

Table 4 shows the effect of acetic acid treatment on the growth of *L*. *monocytogenes* inoculated onto legs. Significant differences (*p* < 0.05) in the *L*. *monocytogenes* populations were observed on legs treated with 2% acetic acid compared to the control samples. After eight days of storage, *L*. *monocytogenes* counts were 1.31 log cycles lower in legs treated with 2% acetic acid than in control ones. Significant reductions in the *L*. *monocytogenes* populations were also observed on legs treated with 1% acetic acid on Days 1, 3, 6 and 8 of storage compared to the control samples. Significant differences were observed between legs treated with 1% and 2% acetic acid on Days 3, 6 and 8. Samples treated with 1% or 2% acetic acid displayed an extended lag phase of *L*. *monocytogenes* and lower counts throughout storage compared with control legs. While *L*. *monocytogenes* grew readily on control legs, growth was slower on acid-dipped legs, particularly those dipped in 2% acetic acid.

**Table 4:** The effect of acetic acid on *Listeria monocytogenes* counts on poultry legs (log CFU/g).

Batch	Days of Storage				
	0	1	3	6	8
Control	4.90 ± 0.20 ^a^	5.53 ± 0.03 ^a^	7.38 ± 0.03 ^a^	7.74 ± 0.01 ^a^	8.77 ± 0.01 ^a^
1% Acetic acid	4.53 ± 0.01 ^ab^	4.38 ± 0.04 ^b^	6.25 ± 0.01 ^b^	7.18 ± 0.06 ^b^	7.88 ± 0.03 ^b^
2% Acetic acid	4.25 ± 0.07 ^b^	4.28 ± 0.01 ^b^	5.72 ± 0.06 ^c^	6.60 ± 0.01 ^c^	7.46 ± 0.01 ^c^

Notes: Mean ± standard deviation. Means within columns followed by the same letter were not significantly different (*p* > 0.05).

The ability of acetic acid to inhibit *L. monocytogenes* can be higher in laboratory media than in foods, according to the results reported by Ahamad and Marth [17]. These authors found that the presence of up 0.1% acetic acid in tryptose broth inhibited the growth of *L*. *monocytogenes* and that the degree of inhibition increased as the temperature of incubation decreased. These authors reported that *L. monocytogenes* growth was suppressed when acetic acid concentrations in the medium were 0.2% at all temperatures tested. According to these authors, acetic acid was the most detrimental to *L. monocytogenes* followed in order by lactic and citric acids. Vermeulen *et al*. [20] also reported that *L. monocytogenes* was not able to grow in nutrient broth with 0.4% acetic acid. Cunningham *et al*. [18] studied the response of *L. monocytogenes* to weak acids, including acetic acid. These authors observed that acetic acid at concentrations of 0.15% in BHI reduced *L. monocytogenes* counts.

George *et al*. [19] also found that acetic acid was more inhibitory to the growth of *L. monocytogenes* than lactic acid in terms of total acid added. According to Farber *et al*. [37], acetic acid increases the minimum pH for the growth of *L. monocytogenes* more than lactic acid. The greater effectiveness of acetic acid could be explained by its lower pKa, giving a greater proportion of acid in the undissociated form [19].

Dorsa *et al*. [13] reported that spray application of 1.5% or 3% acetic acid in beef reduced the levels of *L. innocua* after washing. After, two days of storage at 5 °C, these authors could not detect the growth of *L. innocua*.

Conflicting reports on the efficacy of acetic acid against *L*. *monocytogenes* may be due to variations in the media or food, pH or acid concentration [38]. The efficacy of acetic acid against *Listeria* could be higher in other types of meat, since the pH is lower. Glass and Doyle [39] reported that the *L*. *monocytogenes* grew well on those meat products with a pH value near or above 6.0, while on meats near or below pH 5.0, the organism grew poorly or not at all. Poultry has a higher pH than other types of meat. It should be pointed out that poultry leg muscles have a pH of 6.1, while other parts, like the breast muscles, have lower pH values (5.7–5.9) [40]. This higher pH can explain why poultry supports the growth of *L*. *monocytogenes* better than other meats; for that reason, decreasing the pH with acetic acid treatment could contribute to controlling the growth of *L*. *monocytogenes*.

Other pathogens are also inhibited by acetic acid. Fabrizio *et al*. [26] reported that immersion in a 2% solution of acetic acid reduces *Salmonella* counts by 1.41 log cycles after treatment. Jimenez *et al*. [14] observed *Salmonella* reduction of about 0.4 log cycles when 2.8% acetic acid was applied in poultry.

Moreover, Waterman and Small [41] found that *Salmonella* inoculated onto the surface of pre-acidified ground beef could not survive if the pH on the surface of the beef was 2.61 or lower, but was viable if the surface pH was 3.27. In the present study, although the pH of the acetic acid solution was low, the mean pH value on the legs was 5.08 or 4.39 after treatment, depending on the acid concentration.

It must be highlighted that Uyttendale *et al*. [4] reported that among chicken parts, *L. monocytogenes* was predominantly isolated from chicken legs and chicken wings, the parts that are still partially covered with skin. This pathogen is mainly located on the skin surface of poultry carcasses and, to a lesser, extent in the meat. On the other hand, the higher pH of leg meat may provide more favorable conditions for multiplication of *L. monocytogenes* [1].

*L. monocytogenes* can grow at temperatures as low as 4 °C. Thus, this bacterium is a particular foodborne hazard, because of the ability to replicate, albeit slowly, at refrigeration temperatures [1].

According to Carpenter *et al*. [6], acetic acid displays residual activity to prevent the growth of pathogens. These authors highlighted the role of organic acids in the meat industry, especially the effectiveness of organic acid washes relative to their ability to decontaminate meat tissues and subsequently inhibit the growth of pathogens; thus, organic acids contribute to a total food safety program.

Although treatments with acetic acid did reduce populations of *L*. *monocytogenes* on poultry meat, they were not able to reduce the pathogen to zero levels. Depending on the initial populations of the pathogen, reductions ranging from one log CFU/g may not be sufficient as the only means to improve the overall microbiological safety of poultry carcasses. These results should be considered with caution because of the high *L*. *monocytogenes* load of the artificially inoculated poultry (4.90 log UFC/g), and further research is needed. Overall, the data suggests that acetic acid treatments may be beneficial as part of an overall hazard analysis critical control point (HACCP) approach that can be implemented in order to enhance the microbiological safety and extend the shelf life of poultry meat.

### 3.4. Sensorial Quality

**Figure 2:**
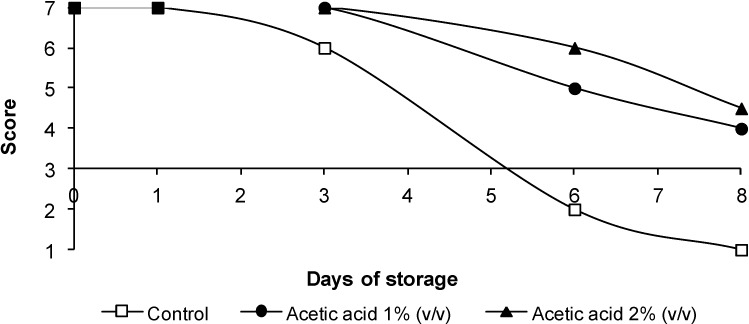
The evolution of color in chicken legs treated with acetic acid. The data are the mean values of six replicates.

**Figure 3:**
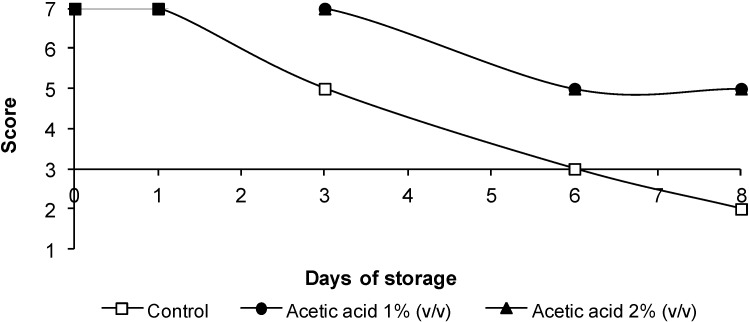
Evolution of odor in chicken legs treated with acetic acid. The data are the mean values of six replicates.

**Figure 4:**
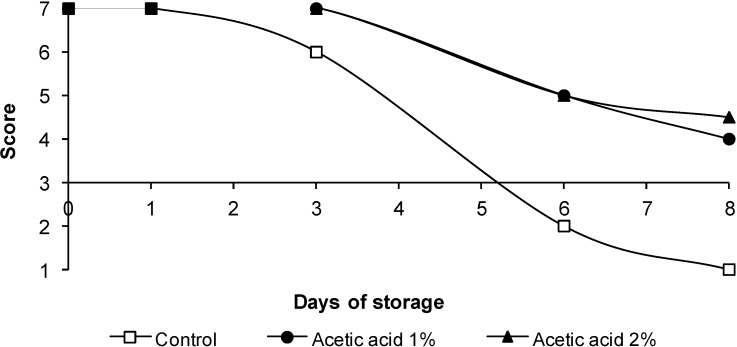
Overall appearance of chicken legs treated with lactic acid and acetic acid. The data are the mean values of six replicates.

The changes in color, odor and overall appearance of the poultry legs are shown in Figure 2, Figure 3 and Figure 4, respectively. Sensory quality was not adversely affected by acetic acid treatment, the scores being observed above six until Day 3. No significant differences (*p* > 0.05) in color were observed between samples treated with acetic acid and control samples until Day 3. After six days of storage, the worst score was obtained by control legs. Control legs were rejected on Day 6. When treatments were compared at Day 6 of storage, treatment with acetic acid reduced (*p* < 0.05) the presence of off-odors compared with the control. The samples treated with 1% or 2% acetic acid were not severely discolored, and unacceptable odors were not detected throughout storage. Consequently, legs receiving treatments with acetic acid remained acceptable until eight days of storage, at least two days longer than control samples.

Acetic acid treatment did not have adverse effects on poultry legs quality characteristics. Other authors have also reported that solutions of organic acids (1%–3%) have no sensorial negative effects in meat when used as a decontaminant [42].

Off-odors were noticed by the panel members when the counts approached 9 log CFU/g. To compare our results with those reported by other authors, the data were transformed to log CFU/cm^2^. It was found that 1 g of skin corresponded to an average of 6.88 cm^2^ of skin. Thus, 9 log CFU/g corresponded to 8.16 log CFU/cm^2^. Other authors have reported spoilage odors in poultry when counts approached 7–8 cfu/cm^2^ [40,43,44].

After six days of storage, mesophiles and psychrotrophs reached populations above 9 log CFU/g in control legs. However, in the legs treated with 1% or 2% acetic acid, mesophile and psychrotroph counts were below 9 log CFU/g after eight days of storage at 4 °C, and signs of spoilage were not detected after eight days of storage. Sensorial scores of treated legs were above those reached by the control legs. Control legs were rejected after six days of storage.

These results agree with those reported by Dickens and Whittemore [12], who did not observe any change in skin appearance due to the 1% acetic acid treatment. Jimenez *et al*. [31] reported, despite the high level attained by microbial populations in poultry treated with acetic acid, that the overall aspect remained acceptable throughout the storage periods. These authors found off-odors in untreated samples, while the treated ones smelt slightly acidic and pleasant. Sakhare *et al*. [15] reported that acetic acid treatment at low concentrations (0.5%) after every step of poultry processing (scalding, defeathering, evisceration) did not affect the appearance of carcasses.

## 4. Conclusions

The shelf life of the samples washed with 1% or 2% acetic acid was extended by at least two days over the control samples washed with distilled water. Legs washed with acetic acid showed a significant (*p* < 0.05) inhibitory effect on *L. monocytogenes* compared to control legs. Sensory quality was not adversely affected by acetic acid.

This study demonstrates that while acetic acid did reduce populations of *L. monocytogenes* on meat, it did not completely inactivate the pathogen. Of the concentrations tested, treatments with 2% acetic acid were the most effective for reducing populations of *L. monocytogenes*.

The application of acetic acid cannot replace the rules of strict hygiene and good manufacturing practice, but it may be used as an additional hurdle contributing to extending the shelf life of raw poultry.
